# Proteomics-based aging clocks in midlife and late-life and risk of dementia

**DOI:** 10.21203/rs.3.rs-5500348/v1

**Published:** 2025-01-13

**Authors:** Sanaz Sedaghat, Saeun Park, Rob Walker, Shuo Wang, Jialing Liu, Timothy Hughes, Behnam Sabayan, Weihong Tang, Josef Coresh, James Pankow, Keenan Walker, Ramon Casanova, Ruth Dubin, Rajat Deo, Jerome Rotter, Alexis Wood, Peter Ganz, Pamela Lutsey, Weihua Guan, Anna Prizment

**Affiliations:** University of Minnesota; University of Minnesota; University of Minnesota; University of Minnesota; University of Minnesota; Wake Forest School of Medicine; Hennepin Healthcare Research Institute; University of Minnesota; New York University Grossman School of Medicine; University of Minnesota; NIH; Department of Biostatistics and Data Science, School of Medicine, Wake Forest University; University of Texas Southwestern Medical Center; University of Pennsylvania; The Lundquist Institute for Biomedical Innovation at Harbor-UCLA Medical Center; Baylor College of Medicine; University of California, San Francisco; University of Minnesota; University of Minnesota; University of Minnesota

**Keywords:** dementia, cognitive function, proteomics-based biological aging clock

## Abstract

**Background:**

Biological age can be quantified by composite proteomic scores, called aging clocks. We investigated whether biological age acceleration (a discrepancy between chronological and biological age) in midlife and late-life is associated with cognitive function and risk of dementia.

**Methods:**

We used two population-based cohort studies: Atherosclerosis Risk in Communities (ARIC) Study and Multi-Ethnic Study of Atherosclerosis (MESA). Proteomics-based aging clocks (PACs) were created in ARIC at midlife (mean age: 58 years, n=11,758) and late-life (mean age: 77 years, n=4,934) using elastic net regression models in two-thirds of dementia-free participants and validated in the remaining one-third of participants. Age acceleration (AA) was calculated as residuals after regressing PACs on chronological age. We validated the midlife PAC in the MESA cohort (mean age: 62 years, n=5,829). We used multivariable linear and Cox proportional hazards regression to assess the association of AA with cognitive function and dementia incidence, respectively.

**Results:**

In ARIC, every five years AA was associated with lower global cognitive function: difference: −0.11, 95% confidence interval (CI): −0.16, −0.06) using midlife AA and difference: −0.17, CI: −0.23, −0.12 using late-life AA. Consistently, midlife AA was associated with higher risk of dementia (hazard ratio [HR]: 1.20 [CI: 1.04, 1.36]) and more prominently when using late-life AA (HR: 2.14 [CI:1.67, 2.73]). Similar findings were observed in the MESA study: every five years AA was associated with lower global cognitive function (difference: −0.08 [CI: −0.14, −0.03]) and higher risk of dementia (HR:1.23 [CI: 1.04, 1.46]).

**Conclusion:**

Accelerated biological age – as defined by the plasma proteome – is associated with lower cognitive function and predicts a higher risk of dementia in midlife and more prominently in late-life.

## Introduction

Dementia is a major cause of death, disability and dependency among older adults worldwide.^1,2^ While age is the most significant risk factor for dementia, it is well recognized that biological aging differs between individuals.^3^ Biological age can deviate from chronological age due to various biological disruptions such as inflammation, oxidative stress, vascular dysfunction, and immune dysregulation.^3^ These biological disturbances typically become more prominent with advancing age, but they vary among individuals and are referred as accelerated biological aging.^4^

Several studies have shown that patients with dementia develop subclinical metabolic changes years before dementia onset.^5,6^ Studies have demonstrated that there are significant differences in plasma biomarker composition between patients with dementia and cognitively intact individuals.^5,7^ This finding has stimulated various lines of research to build up biological aging clocks that can predict future decline in brain structural and functional integrity.^8^ Specifically, biological aging processes can be quantified using composite metrics referred to as aging clocks using plasma protein biomarkers.^3^

Proteomics-based aging clocks (PACs) are promising biomarkers of aging because proteins expression changes with advancing age and they exert biological functions which can be potentially modified by lifestyle and pharmaceutical interventions.^3,9^ In this study, we created a proteomics-based aging biological clock across two stages of life namely, midlife and late-life, then tested the hypotheses that biological age acceleration would be associated with lower global and domain-specific cognitive function as well as greater risk of developing dementia. We performed this study in the setting of two large prospective population-based cohort studies. PACs were created and tested in the Atherosclerosis Risk in the Communities (ARIC) study, a cohort of mostly White and Black men and women with plasma proteomics data that have been collected over 20 years of follow-up, and then validated and replicated in the Multi-Ethnic Study of Atherosclerosis (MESA), a prospective cohort including ethnically diverse participants identifying as Black, White, Asian, and Hispanic.

## Methods

### The ARIC study population

The ARIC study is a longstanding prospective cohort of 15,792 participants (45–64 years old) started in 1987–1989 (Visit 1).^10–12^ Participants were recruited from four communities in the United States (suburban Minneapolis, MN; Washington County, MD; Forsyth County, NC; and Jackson, MS).^13^ Participants have been re-invited for follow-up visits, including Visit 2 (1990–1992), Visit 3 (1993–1995), Visit 4 (1996–1998), Visit 5 (2011–2013), Visit 6 (2016–2017) and Visit 7 (2018–2019), of relevance to this analysis. We included 11,758 participants with information on cognitive function and protein measurements at Visit 2 (midlife) and 4,934 participants at Visit 5 (late life) (Figures S1A & 1B) to train midlife and late-life PACs and analyze their association with incident dementia.

We also investigated the association between PACs and cognitive function. We included 5,123 participants at Visit 5 (late-life) who had available information on both cognitive function and proteins (Figure S2). There were 4,783 participants with protein data at Visit 2 (midlife) and cognitive function data at Visit 5 (Figure S2).

The study was approved by each site’s institutional review board, and written informed consent was signed by all participants (or proxies, when required).

### Proteomics measurement

In ARIC, plasma proteins have been measured using a SOMAmer (Slow Off-rate Modified Aptamers)-based assay called SomaScan (V4.0) (SomaLogic, Inc., USA)^14^ in stored blood samples collected at Visit 2 (midlife) and Visit 5 (late-life). The SomaScan platform uses single-stranded DNA-based protein-bound aptamers to capture conformational protein epitopes.^14,15^ The aptamers are mapped to unique proteins using the Universal Protein Resource (UniProt) databases.^15,16^ Approximately 5000 proteins (4955 aptamers and 4712 unique proteins) measured at midlife and late-life underwent SomaScan standardization and normalization processing as previously described.^17,18^ Briefly, hybridization control normalization was applied to each sample to correct systematic biases, followed by median signal normalization to eliminate sample or assay biases within plates. Based on global reference plate-to-plate variations were assessed and protein analytes with calibration factor ±0.4 (the median calibration factor) were excluded from all analyses. This process was used to ensure minimal batch effect and absence of systematic biases when using proteins from different visits longitudinally. To correct for skewness, all aptamer measures were log base 2 transformed. We ran blind split-sample duplicate plasma aliquots and observed median coefficients variation of 6% and 7%, and median Pearson correlations of 0.93 and 0.96 at midlife (Visit 2) and late-life (Visit 5), respectively.

### Cognitive function assessment

All participants completed a 60-minute comprehensive neuropsychological assessment administered by trained and certified psychometrists at Visit 5 (late-life). The measures are well-validated and standardized instruments, which assess multiple domains of cognition including memory, executive function/processing speed and global cognitive function.^19^ The test battery includes: Memory domain: Delayed Word Recall Test: a test of verbal memory requiring recall of a word list after a short delay (score range 0–10). Logical Memory I and II: from the Wechsler Memory Scale-Revised (WMS-R) is a test of immediate (Logical Memory I) and delayed (Logical Memory II) memory. Executive function/processing speed domain: Trail Making Test Part A: In Trail Making A participants are asked to draw a line connecting circles numbered 1 to 25 that are randomly distributed on the page as fast as possible. Digit Span Backwards: a test of attention in which participants state a series of digits backward. Digit Symbol Substitution Test: a subtest of the Wechsler Adult Intelligence Scale-Revised involving timed translation of numbers to symbols in 90 seconds using a key, which measures psychomotor performance (score range 0–93). Word Fluency Test: combined total of correct words produced beginning with F, A and S. Category Fluency Test: participant is asked to spontaneously generate words from a specific category (in this test, animals).^19,20^ For global cognitive function assessment, we included all the aforementioned tests as well as Boston Naming Test: a test of language in which participants name common objects from pictures. To create scores for each cognitive domain and global cognitive function, we used principal component analysis (PCA) to derive three cognitive function scores for memory, executive function/processing speed, and global cognition (combination of all cognitive domains). Before PCA analysis, cognitive function test scores were checked for normal distribution. Participants with no cognitive function scores were excluded (N=60). Trails A test scores were inversed so that low test scores indicate poorer cognitive function and higher test scores indicate better cognitive function for all tests. Imputation via mean was used to impute any missing values for cognitive tests. Next, Z-scores were calculated for all cognitive test scores and PCA was conducted to create three distinct factor scores for memory, executive function/processing speed, and global cognition. Percentages of variance explained by PCA factors for each cognitive domain are compiled in Table S1.

### Dementia incidence

Dementia incidencewas assessed using well-validated, standardized battery of cognitive measures supplemented by dementia surveillance in between visits, and hospital discharge or death certificate.^19,21–24^ In short, all participants underwent a 3-instrument cognitive assessment at Visit 2, Visit 3 and Visit 4. The 3-instrument cognitive testing was repeated in a subset at the ARIC-MRI examination in 2004–2006 (Jackson and Forsyth County sites only); and again, in all participants who took part in in-person assessments at Visits 5, 6 and 7 as part of the ARIC-NCS (NeuroCognitive Study). From Visit 5 onwards, those unwilling or unable to attend the in-clinic assessment were invited for an in-person assessment in their home or long-term care facility. If they did not take place in-person in visit 5 they were offered a modified telephone interview for cognitive status (TICSm). Beginning in 2012, participants were screened for dementia on annual or semi-annual cohort follow-up calls using the Six-Item Screener, then for those with indication of impaired cognition the AD8 was conducted with proxies. The data was supplemented by ICD codes for dementia identified through surveillance of hospital discharges or death certificates. The information on dementia was reviewed according to a standard protocol by the ARIC Neurocognitive Classification Committee. The dementia onset was the earliest date determined by in-person visit assessment, dementia surveillance, hospital discharge, or death certificate code. When dementia was identified through an informant interview, hospitalization record, or death certificate, the date of diagnosis was estimated to occur 180 days before the documented incident or interview.^19^ Follow-up time was defined as the number of days from the participant’s baseline exam to the date of incident dementia event, loss to follow-up, death from another cause, or censoring date at December 31st, 2019, whichever occurred first.

### Other covariates

All covariates were assessed at visits where proteins were measured (ARIC: Visit 2, midlife and Visit 5, late-life). Race was self-reported and was classified as Black or White. Cigarette smoking and education were assessed using questionnaires and were categorized as current, former, or never users for smoking status and less than completed high school, high school equivalent, and greater than high school for education (measured at Visit 1). Diabetes was defined as self-reported history of physician diagnosis, antidiabetic medication use during the past 2 weeks, fasting blood glucose level ≥ 126 mg/dL, or nonfasting blood glucose level ≥ 200 mg/dL. Trained technicians measured blood pressure with participants sitting after 5-minute rest. Blood pressure was measured three times using and the average of the last two readings was recorded. Hypertension was defined as systolic blood pressure greater than 140 mm Hg or diastolic blood pressure greater than 90 mm Hg or using antihypertensive medications. Plasma total cholesterol and creatinine and cystatin C were measured using enzymatic methods. Estimated glomerular filtration rate (eGFR) was calculated using CKD-EPI 2021 equation.^25^ Genotyping for APOE was performed by TaqMan assay (Applied Biosystems, Foster City, Calif).

### Validation of PACs in MESA cohort

The MESA cohort included 6,814 men and women aged between 45 and 84 who identified their race/ethnicity as White, Black, Chinese, or Hispanic/Latino who had no history of clinical cardiovascular disease (CVD) at enrollment. Participants were recruited from Baltimore, Maryland; Chicago, Illinois; Forsyth County, North Carolina; Los Angeles County, California; New York, New York; and St. Paul, Minnesota. Enrollment and the baseline exam (Exam 1) occurred between 2000 and 2002. Participants have been re-invited for follow-up visits, including Exam 2 (2002–2004), Exam 3 (2004–2005), Exam 4 (2005–2007), Exam 5 (2010–2011), Exam 6 (2016–2018), and Exam 7 (2022–2024).^26^ We included 4,057 participants with protein measurements at Exam 1 and cognitive function information at Exam 5 and 5,829 participants with both protein measurements and information on dementia status (Figures S3). The institutional review boards at all participating sites approved the study, and all participants provided written informed consent.

Proteins have been measured using a newer SomaScan version (V4.1) including 7000 proteins. This version contains all 5000 proteins from the previous version of SomaScan assay that was used in ARIC. We used the same quality checks and protocols as in ARIC. To confirm similar measurements of proteins in MESA and ARIC cohorts, we compared distribution and summary statistics of aptamers between the studies; 4 random aptamers (protein units) presented in Figure S4. Cognitive function was measured in research setting at Exam 5 (2010–2011). The battery includes Cognitive Abilities Screening Instrument (CASI), digit symbol coding, forward digit span, and backwards digit span. General instructions for the cognitive examination were translated into Spanish and Mandarin Chinese and then independently back-translated by native speakers and pretested.^27^ We used individual cognitive tests in MESA. Incident dementia was ascertained through ICD-9 and ICD-10 codes in medical records for hospitalizations reported during follow-up interviews as well as in dementia death certificates. The codes used to define dementia have been listed previously.^28^ Follow-up time was defined as the number of days from the participant’s baseline exam to the date of incident dementia event, loss to follow-up, death from another cause, or censoring date at December 31st, 2018, whichever occurred first.

### Statistical analysis

#### Proteomic aging clocks (PACs) and age acceleration

**Figure 1:**
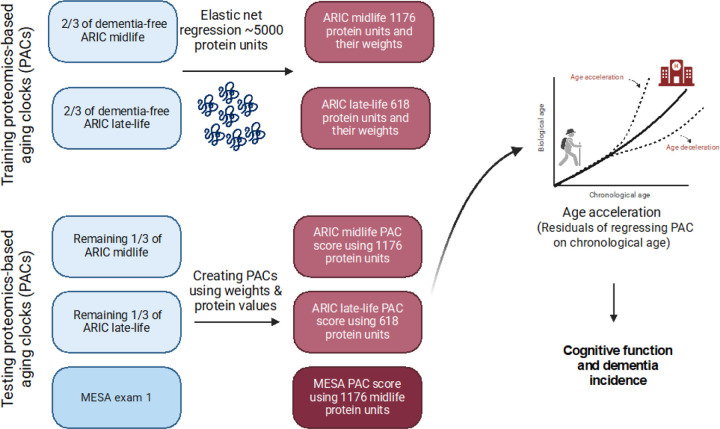
Proteomics-based aging clock (PAC) training and validation. Weights are regression coefficients from the elastic net regression models.

We created and trained the PACs in the ARIC cohort. To construct midlife and late-life ARIC dementia-free PACs, we randomly selected two-thirds of participants who remained free of dementia until 2019 at each visit and used them as the training set at the corresponding visits. The remaining one-third of participants who remained free of dementia until 2019 were used as the test set at the corresponding visits (Figure S1A and B). Using the training set, we applied elastic net regression to train the ARIC dementia-free PACs against age as a weighted sum of aptamers: , where is the level of the *ith* aptamer.^29^ Lambda value was selected based on 10-fold cross-validation in the training set. This resulted in selection of 1176 aptamers in ARIC midlife and 618 aptamers in ARIC late-life participants (Appendix Table 1 and 2). We internally validated the ARIC dementia-free PACs by examining their correlation with age in the remaining participants at the corresponding visits. To capture PACs’ effect independent of age, we created proteomic age acceleration for each PAC as residuals by regressing PAC on chronological age in the remaining participants after excluding the training set at the corresponding visits. A positive value of age acceleration suggests that the proteomic age tends to be older than the person’s chronological age (Figure 1). To understand the combination of proteins contributed to midlife and late-life PACs, we took a closer look at the overlapping aptamers (Figure S5, Appendix Table 3). There were 270 overlapping aptamers between midlife and late-life. At both timepoints we selected the top 10 proteins based on effect estimates for presentation herein. Among them, 6 were both at midlife and late-life, so a total of 14 proteins are presented Table S2).

#### Age acceleration and cognitive function

Multivariate linear regression was used to calculate adjusted effect estimates and 95% confidence intervals (CI) for age acceleration (per 5 years) and global, and cognitive function domain scores. The analyses were ran using ARIC Visit 2 (midlife) and Visit 5 (late-life) age acceleration measures and cognitive function assessed at Visit 5 (late-life). For all analyses, we ran two models: first model adjusted for chronological age, sex, race/ethnicity, study center and the second model additionally adjusted for education, body mass index (BMI), smoking status, hypertension, diabetes status, cholesterol, and eGFR.

#### Age acceleration and dementia

We used Cox proportional hazards regression models to examine the association of age acceleration (per 5 years) with incident dementia. Analyses include participants from ARIC Visit 2 (midlife) and ARIC Visit 5 (late-life) to the date of incident dementia event, loss to follow-up, death from another cause, or end of follow-up. We ran the analyses in the remaining participants after excluding the training set at each visit. Because the remaining set included those who had dementia in ARIC, we applied a case-cohort weighting scheme employing Barlow’s method to account for the imbalance of dementia-free (one-third) and dementia (100%) participants.^30^ Following the case-cohort analysis method, we created a “subcohort” which consisted of the participants who were free of dementia in the remaining set and one-third randomly selected participants who developed dementia during follow-up, since the training and test sets split was 2:1. We ran all analyses in two adjustment models as mentioned before. The proportional hazards assumptions were not violated, as assessed by visual inspection of the survival curves and assessing Schoenfeld residuals.

#### External validation in MESA

PACs were computed by multiplying the concentration of log 2-transformed proteins at Exam 1 by regression coefficients (weights) calculated in ARIC. The distribution of age at ARIC midlife and MESA Exam 1 are similar, while the late-life ARIC population is on average older than the MESA Exam 1 participants; therefore, we used PAC created at ARIC Visit 2 (midlife) as our primary clock for replication in MESA and PAC at ARIC Visit 5 (late-life) as a secondary clock (Figure 1). The performance of the PACs were tested by (1) plotting the chronological age against PACs and (2) calculating median absolute error and Pearson correlation (r) with chronological age between PAC and chronological age. Ideally, r should be > 0.7, see Table S3 for r values. We then calculated age acceleration as described before, i.e., as residuals of PAC regressed o chronological age.

Multivariate linear regression was used to calculate adjusted effect estimates and 95% confidence intervals (CI) for age acceleration and individual cognitive function scores. We used age acceleration calculated at MESA Exam 1 (2000–2002) and cognitive function at Exam 5 (2010–2011). We used Cox proportional hazard regression models to examine the association of age acceleration (per 5 years) with the incidence of dementia. All analyses were done in two models adjusted for similar covariates as ARIC cohort. We repeated the analyses in MESA using proteins and coefficients based on ARIC late-life PACs (instead of ARIC midlife) in association with cognitive function and dementia incidence.

#### Sensitivity analyses in ARIC cohort

To better understand the differences between midlife and late-life PACs, we re-created a PAC in ARIC late-life by using proteins selection and regression coefficients (weights) from ARIC midlife and applying them to protein levels from late-life. To see if the results are different based on race and *APOE* ε4 carriership (carrying 1 or 2 ε4 alleles), we stratified based on race and *APOE* ε4 allele carriership (carrying 1 or 2 ε4 alleles compared with no ε4 allele). We also excluded participants with prevalent stroke to confirm that history of clinical stroke does not change the findings.

## Results

Baseline characteristics of participants in midlife and late-life cohorts in ARIC as well as MESA are presented in Table 1. Participant’s characteristics in those with and without incident dementia are presented in Table S4A and B. In addition, baseline characteristics of participants in the subset of those with cognitive function data are presented in Table S5. Age acceleration ranged in ARIC from −11.5 to 16.8 years at midlife and −7.4 to 12.5 years at late-life. In MESA, age acceleration ranged from −12.4 to 17.0 years. For dementia incidence, median follow-up time since ARIC Visit 2 (midlife) and Visit 5 (late life) were 21 (interquartile range: 11) and 6 (interquartile range: 3) years, respectively. In MESA median follow-up time was 17 years (Interquartile range: 5).

### Associations of age acceleration with cognitive function and dementia incidence

In fully adjusted model, each 5-year age acceleration at midlife was associated with lower late-life executive function (standardized difference: −0.14 [95% CI: −0.19, −0.09]) and global cognitive function (difference: −0.11 [95% CI: −0.16, −0.06]). There was no association between age acceleration and memory function. When using age acceleration at late-life, each 5-year age acceleration was cross-sectionally associated with lower memory (difference: −0.11 [95% CI: −0.18, −0.05]), executive function (difference: −0.19 [95% CI: −0.24, −0.14]) and global cognitive function (difference: −0.17 [95% CI: −0.23, −0.12]) (Table 2).

**Figure 2:**
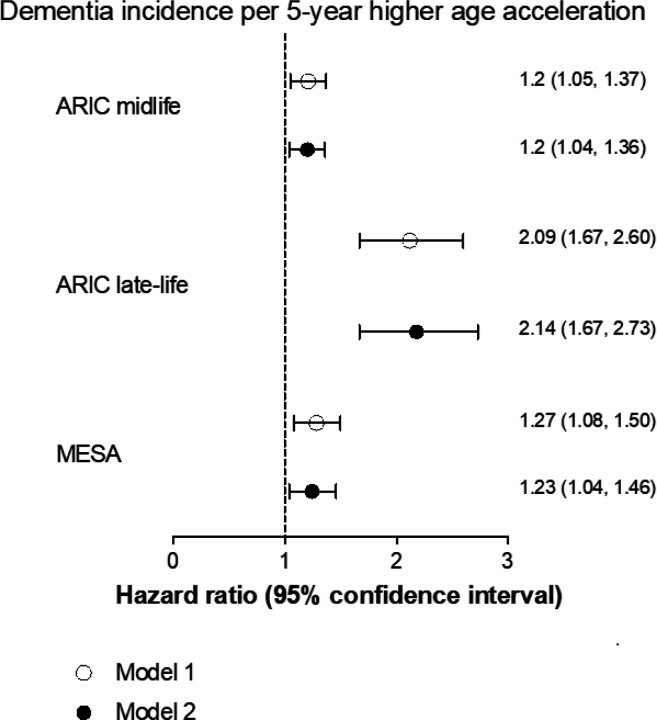
Association between age acceleration per 5 years (5-year discrepancy between chronological and biological age) and dementia incidence. Model 1 is adjusted for chronological age, sex, race/ethnicity, and study center Model 2 additionally adjusted for education, BMI, smoking status, systolic blood pressure, diabetes status, cholesterol, and estimated glomerular filtration rate. Number of cases: ARIC midlife=2,251; ARIC late-life=707; MESA=506.

In the fully adjusted model, at ARIC midlife, each 5-year age acceleration was associated with 20% higher risk of incident dementia (HR: 1.20, 95%CI: 1.04, 1.36). Each 5-year age acceleration at ARIC late-life was more prominently associated with dementia risk with a HR of 2.14 [95%CI: 1.67, 2.73] (Figure 2).

### Validation in MESA

Similar to ARIC findings, in MESA, age acceleration was prospectively associated with lower cognitive function performance (Table 3). Similarly, each 5-year age acceleration (using ARIC midlife PAC) was associated with 1.23 [95%CI: 1.04, 1.46] higher hazard of dementia (Figure 2). When using ARIC late-life PAC (proteins and coefficients based on ARIC late-life PAC) in MESA, we observed stronger effect estimates with dementia risk (1.61 [95%CI: 1.29, 2.01]) (Table S6) and similar results with cognitive function (Table S7).

### Sensitivity analyses in ARIC cohort

Using the selection of proteins and regression coefficients from ARIC midlife and protein levels from ARIC late-life, the effect estimates for late-life PAC lie between effect estimates from midlife and late-life (Table S8). Stratifying by race (Table S9) *APOE* ε4 allele carriership (Table S10) and excluding those with prevalent stroke (data not shown) didn’t change the findings.

## Discussion

In this study, we show that higher biological age acceleration, which reflects the deviation of biological age from chronological age, is associated with lower performance in cognitive tests particularly in relation to executive function and processing speed and higher risk of developing dementia. The associations were independent of chronological age, demographic and cardiovascular risk factors. Our results indicate that PACs can be considered as a tool to identify individuals at risk for cognitive impairment and developing dementia in future.

Prior studies investigated roles of different types of biological clocks in predicting future risk of cognitive impairment and dementia.^31^ For instance, various DNA methylation epigenetic clocks have been tested by multiple studies as marker for advanced cognitive aging and dementia incidence.^32–35^ Combining the data in a systematic review and meta-analysis, Zhou et al. showed that majority of these studies did not show a significant association and concluded that there is insufficient evidence to indicate that epigenetic aging can serve as a valid biomarker to individuals at risk for cognitive impairment and dementia.^31^ Such mixed and inconclusive results could be due to using a heterogenous group of DNA methylation aging clocks.^36^ Prior studies have shown that proteins have the potential to serve as metrics for quantifying biological aging, potentially outperforming DNA methylation aging clocks.^37,38^ Proteins can be more accurately measured than methylation CpG sites and are closer to phenotypic expression.^3^ Moreover, in clinical contexts, proteins are more useful, as medical professionals routinely rely on plasma proteins as biomarkers for diagnosing medical conditions, predicting outcomes, and assessing treatment efficacy. Sathyan et al. showed that a higher age acceleration, using proteomics clocks, predicts risk of motor cognitive risk syndrome, a pre-dementia syndrome characterized by slow gait and subjective cognitive concerns.^39^ In the current study, we developed multiple PACs and showed that these clocks predict risk of dementia in both midlife and late-life in two separate cohorts consisting of different racial and ethnic groups. Future studies with focus on application of these clocks in clinical settings for prediction and patient risk stratification are warranted to bring the scientific evidence closer to clinical practice.

Notably, we observed a stronger association with risk of dementia when using clocks developed in late-life as opposed to those at midlife. This finding might reflect the dynamic nature of biological markers across the lifespan and highlight the importance of considering age-specific changes in disease prediction models. The stronger predictive value of PACs in older age can be due to the fact that there is a greater variability in protein levels in older age, potentially making it a more effective tool for discerning differences. In addition, with aging there is a progressive accumulation of molecular alterations, such as increased oxidative stress, impaired protein clearance mechanisms, and chronic inflammation.^3,9^ These age-related changes may contribute to distinct proteomic signatures that can better reflect the evolving pathological processes underlying dementia development in later life.^40^ Another possibility is that late-life PACs are closer to the onset of dementia, making them potentially superior predictors. While proteomic alterations in midlife may reflect early pathological changes associated with dementia, they may not fully capture the complexity of the disease cascade that unfolds over several decades. To investigate whether the protein selection at older age is the driving factor, we constructed a PACs in older age using identified proteins from midlife, instead of those selected at late-life, then evaluated the association in the ARIC test set. While we observed a decline in the magnitude of effect estimates for dementia risk using this clock, the estimates remained stronger than midlife estimates, suggesting that the stronger late-life estimates are not solely due to the combination of proteins in the biological clock at late-life. As the effect estimate was still stronger than the midlife biological clock, it is possible that both factors mentioned earlier contribute to the difference between midlife and late-life estimates.

Dementia has a long preclinical phase which typically takes decades to manifest as cognitive function impairments. To explore whether the PACs can be used to predict dementia risk at earlier stages of cognitive decline, we evaluated the association of the clocks with cognitive function. While, both midlife and late-life clocks were associated with decrease in global cognition and executive function, only late-life clock was associated with memory function. A possible explanation could be that usually impairment in executive function precedes memory impairments before full-blown dementia is presented.^41,42^

Our study had several strengths including large sample size, representation of different racial and ethnic groups, external validation of the results in an independent cohort, multiple assessment of proteomics over time, longitudinal data collection spanning midlife and late-life and availability of detailed information about patient characteristics and potential confounders. We also acknowledge several limitations of this study. First, information on dementia subtypes was not available in all participants and there were differences in the methods for ascertaining dementia between the two cohorts. In addition, ARIC and MESA used different cognitive tests to assess global and domain specific cognitive function. Nevertheless, we observed similar associations with both midlife and late-life clocks derived from ARIC study in MESA cohort, underscoring the robustness of our findings. Although we accounted for multiple demographic and cardiovascular factors, as well as *APOE* ε4 status in our analyses, given the observational nature of this study we cannot rule out the possible effect of unmeasured confounders in the observed associations. Third, PACs are limited in identifying proteins responsible for dementia risk that aren’t age-related.

This study provides new evidence regarding the utility of PACs for predicting dementia and cognitive impairment. The robust link between proteomic profiles and future dementia risk, particularly in late life, has a potential for translation in clinical practice for early detection of high risk individuals and implementation of preventive strategies in individuals at risk.

## Supplementary Material

Supplement 1

## Figures and Tables

**Table 1.** Characteristics at midlife (1990–1992) and late-life (2011–2013) in ARIC and Exam 1 (2000–2002) in MESA.

	ARIC		MESA
	Midlife	Late-life	
Characteristics	Total (N=5,420)	Total (N=2,116)	Total (N=5,829)
**Demographics**			
Mean age, years (SD)	58.3 (5.7)	76.8 (5.3)	62.1 (10.3)

Gender (N Female, %)	3,088 (57.0)	1,196 (56.5)	3,033 (52.0)
Race Group (N, %)			
Black	1,322 (24.4)	420 (19.8)	1,525 (26.1)
White	4,098 (75.6)	1,696 (80.2)	2,301 (39.5)
Hispanic/Latino	-	-	1,305 (22.4)
Chinese	-	-	698 (12.0)
Education (N, %)			
Less than High School	1,283 (23.7)	324 (15.3)	1,045 (18.0)
High School Equivalent	2,264 (41.8)	914 (43.3)	1,068 (18.4)
Greater than High School	1,866 (34.5)	874 (41.4)	3,698 (63.6)

**Lifestyle/Comorbidity Factors**			
Mean BMI, kg/m^2^ (SD)	28.1 (5.4)	28.5 (5.7)	28.4 (5.5)
Smoking Status (N, %)			
Current Smoker	1,147 (21.2)	128 (6.7)	762 (13.1)
Former Smoker	1,995 (36.9)	978 (51.5)	2,154 (37.1)
Never Smoked	2,270 (41.9)	794 (41.8)	2,896 (49.8)
Hypertension (N, %)	2,052 (38.0)	1,585 (75.7)	2,590 (44.4)
Diabetes (N, %)	868 (16.1)	685 (33.2)	1,572 (27.0)
Mean eGFR, mL/min/1.73 m^2^ (SD)	96.2 (13.7)	70.8 (17.7)	74.4 (16.6)
Mean Cholesterol, mg/dL (SD)	211.8 (40.9)	180.2 (41.2)	194.3 (35.9)

Abbreviations: Standard deviation= SD, body mass index= BMI, N= number

**Table 2.** Association between 5-year age acceleration at midlife and late-life and cognitive function Z-scores at late-life – ARIC midlife (1990–1992) and late-life (2011–2013).

	Midlife (N=4,783)			Late-life (N=5,123)		
Cognitive Function Measures	Model 1*	Model 2**	Model 2	Model 1*	Model 2**	Model 2
	Difference (95% CI)	Difference (95% CI)	P-value	Difference (95% CI)	Difference (95% CI)	P-value
**Principal Component Analysis Factors**						
Memory	−0.08 (−0.14, −0.02)	−0.05 (−0.11, 0.02)	0.1419	−0.17 (−0.23, −0.11)	−0.11 (−0.18, −0.05)	0.0008
Executive Function	−0.17 (−0.22, −0.12)	−0.14 (−0.19, −0.09)	<.0001	−0.27 (−0.32, −0.22)	−0.19 (−0.24, −0.14)	<0.0001
Global Cognitive Function	−0.14 (−0.20, −0.09)	−0.11 (−0.16, −0.06)	<.0001	−0.25 (−0.30, −0.20)	−0.17 (−0.23, −0.12)	<0.0001

* Model 1 is adjusted for chronological age at mid/late-life, sex, and race-center

** Model 2 is additionally adjusted for education, BMI, smoking status, systolic blood pressure, diabetes status, cholesterol, and estimated glomerular filtration rate.

**Table 3.** Association between 5-year age acceleration at MESA Exam 1 (2000–2002) and cognitive function Z-scores at Exam 5 (2010–2011) - MESA.

	MESA (N=4,057)		
Cognitive Function Measures	Model 1**	Model 2***	Model 2
	Difference (95% CI)	Difference (95% CI)	P-value
CASI Score	−0.13 (−0.19, −0.07)	−0.08 (−0.14, −0.03)	0.004
Digit Span Forward	−0.10 (−0.15, −0.04)	−0.06 (−0.12, −0.01)	0.028
Digit Span Backward	−0.14 (−0.20, −0.08)	−0.09 (−0.15, −0.04)	0.0012
Digit Symbol Substitution	−0.14 (−0.19, −0.09)	−0.07 (−0.12, −0.03)	0.0025

* Exam 1 age acceleration is calculated using proteins and coefficients from ARIC midlife.

** Model 1 is adjusted for chronological age, sex, race/ethnicity, and study center

*** Model 2 is additionally adjusted for education, BMI, smoking status, systolic blood pressure, diabetes status, cholesterol, and estimated glomerular filtration rate.

Abbreviations: CASI= The Cognitive Abilities Screening Instrument

## Abbreviations

CASI: The Cognitive Abilities Screening Instrument
