# Supplementary material for: Proteomics-based aging clocks in midlife and late-life and risk of dementia
Source: Res Sq. 2025 Jan 13:rs.3.rs-5500348. Preprint. [Version 1] doi: 10.21203/rs.3.rs-5500348/v1 (PMC11774457; doi:10.21203/rs.3.rs-5500348/v1)
Supplement: Supplement 1 [file NIHPPRS5500348v1-supplement-1.pdf]

Supplementary Files

This is a list of supplementary files associated with this preprint. Click to download.

- SupplementAptamersusedtoconstructPACmidlifelatelife.xlsx
- Supplementaldocument.docx
